# Supplementary material for: Atomically thin quantum light-emitting diodes
Source: Nat Commun. 2016 Sep 26;7:12978. doi: 10.1038/ncomms12978 (PMC5052681; doi:10.1038/ncomms12978)
Supplement: Supplementary Information — Supplementary Figures 1-16, Supplementary Notes 1-3 and Supplementary References [file ncomms12978-s1.pdf]

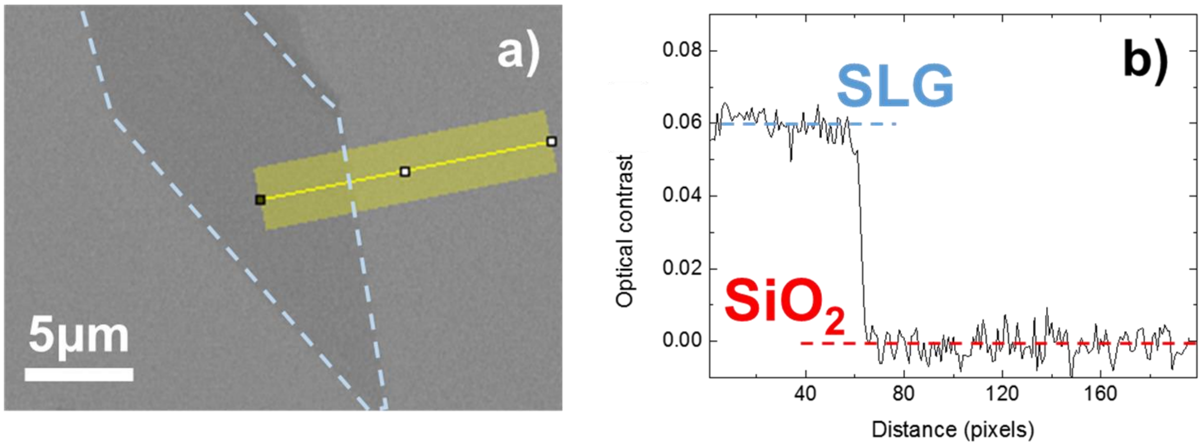

Supplementary Figure 1: Characterisation of single layer graphene (SLG) via optical contrast. a) optical picture of SLG on Si/SiO_2. Dashed area highlights SLG. The yellow line indicates pixels where contrast is measured. b) optical contrast along the yellow line.

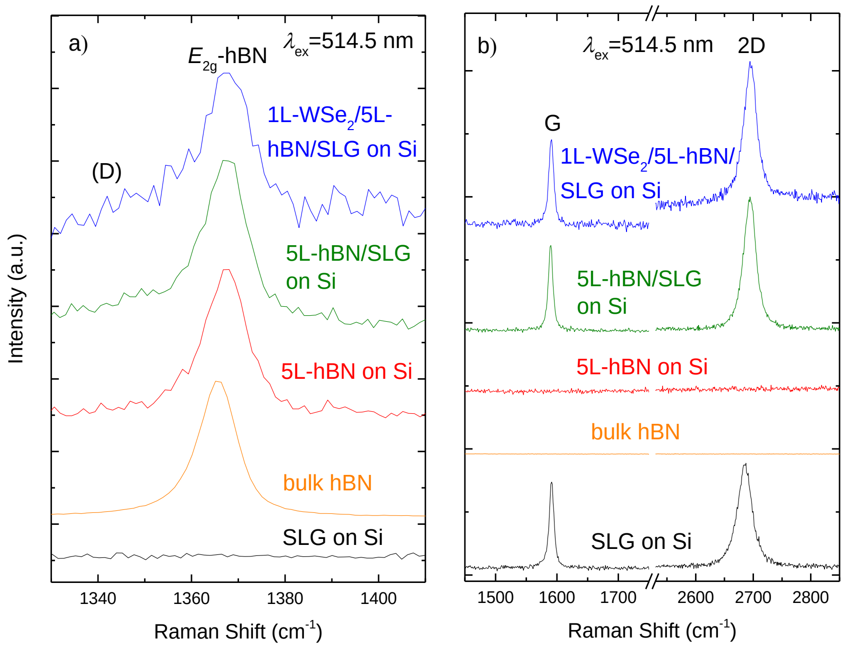

Supplementary Figure 2: Raman of hBN and SLG at different stages of WSe_2 device fabrication. Raman spectra of SLG on Si/SiO_2 (black curve), 5L-hBN on Si/SiO_2 (red curve), 5L-hBN/SLG (green curve), and 1L-WSe_2/5L-hBN/SLG (blue curve), measured at 514.5 nm, with a) and b) showing the hBN and SLG signatures respectively.

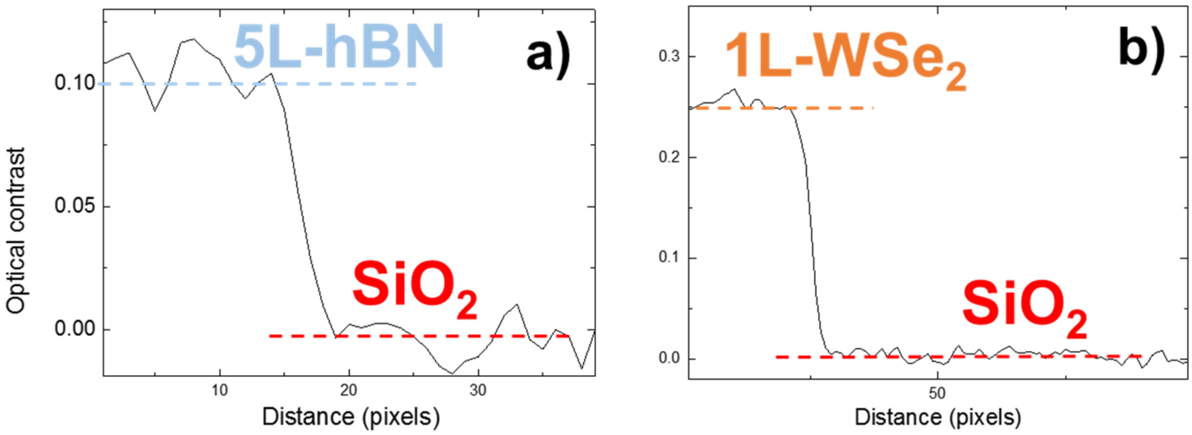

Supplementary Figure 3: Characterisation of few layer hBN via optical contrast. a) optical contrast of 5L-hBN at 580 nm. b) optical contrast of 1L-WSe_2 flake in the green channel. Contrast is ~25%.

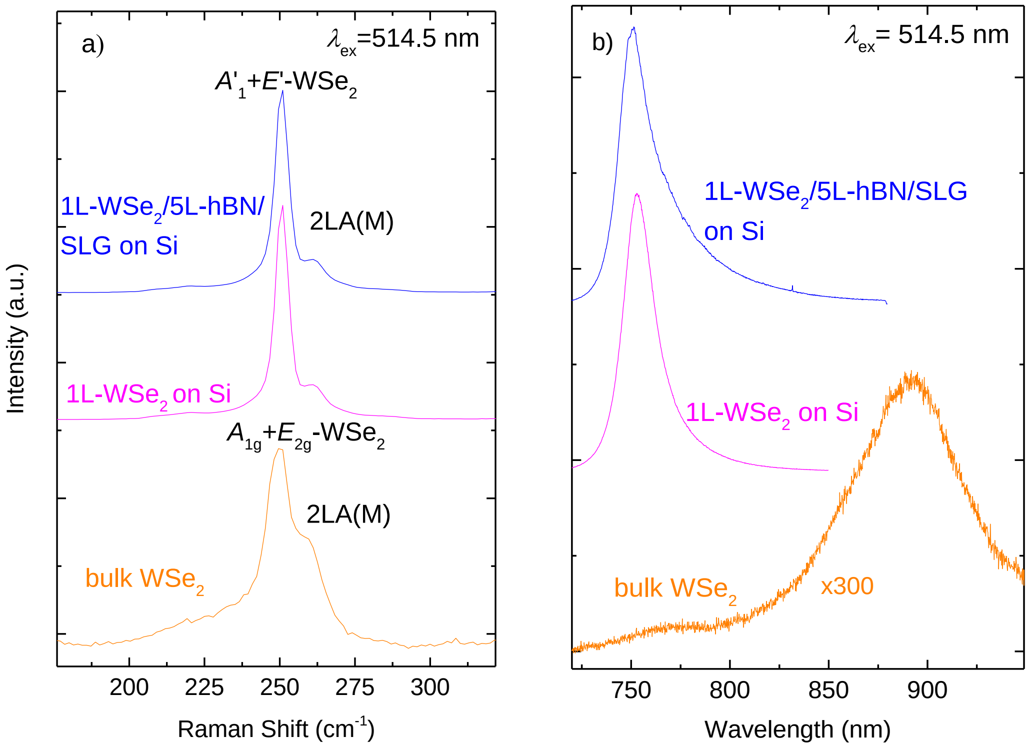

Supplementary Figure 4: WSe_2 Raman and PL on different materials. Comparison of (a) Raman and (b) PL spectra of 1L-WSe_2 on Si/SiO_2 (pink curve) and 1L-WSe_2/5L-hBN/SLG on Si/SiO_2 (blue curve). Excitation wavelength 514.5 nm.

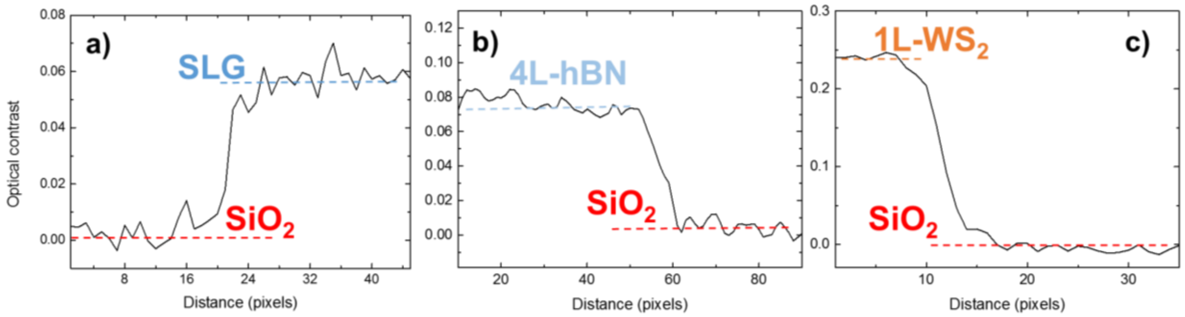

Supplementary Figure 5: Optical contrast of SLG, 4L-hBN and 1L-WS_2. a) SLG, with optical contrast ~5.5%; b) 4L-hBN, with optical contrast ~7.4%, c) 1L-WS_2, with contrast ~24%.

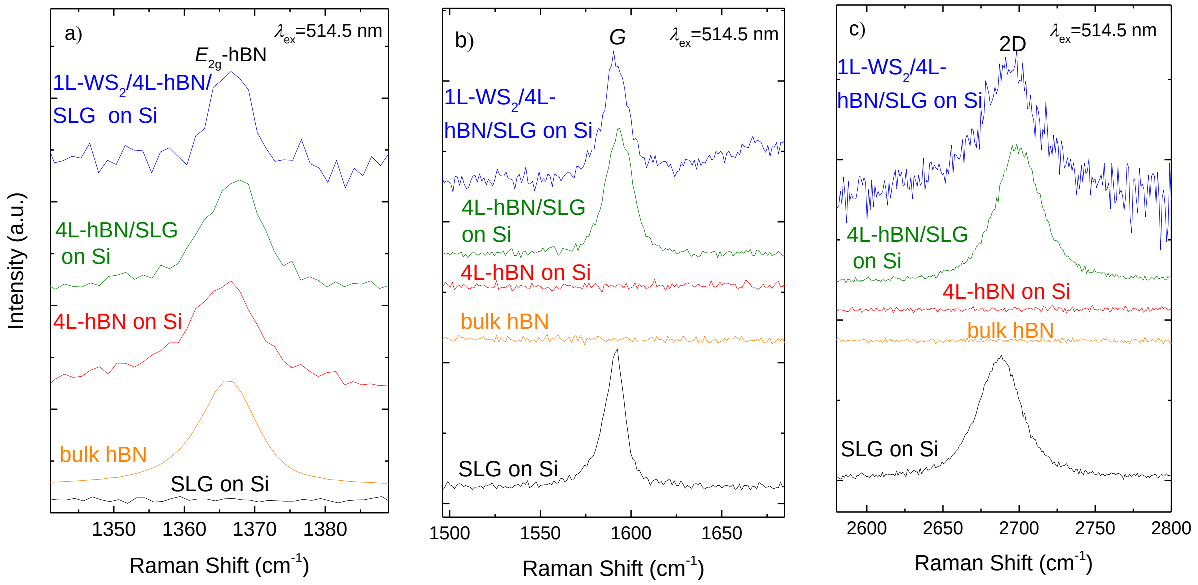

Supplementary Figure 6: Raman of hBN and SLG at different stages of WS_2 device fabrication. Raman spectra of SLG on Si/SiO_2 (black curve), 4L-hBN on Si/SiO_2 (red curve), 4L-hBN/SLG (green curve), and 1L-WS_2/4L-hBN/SLG (blue curve).

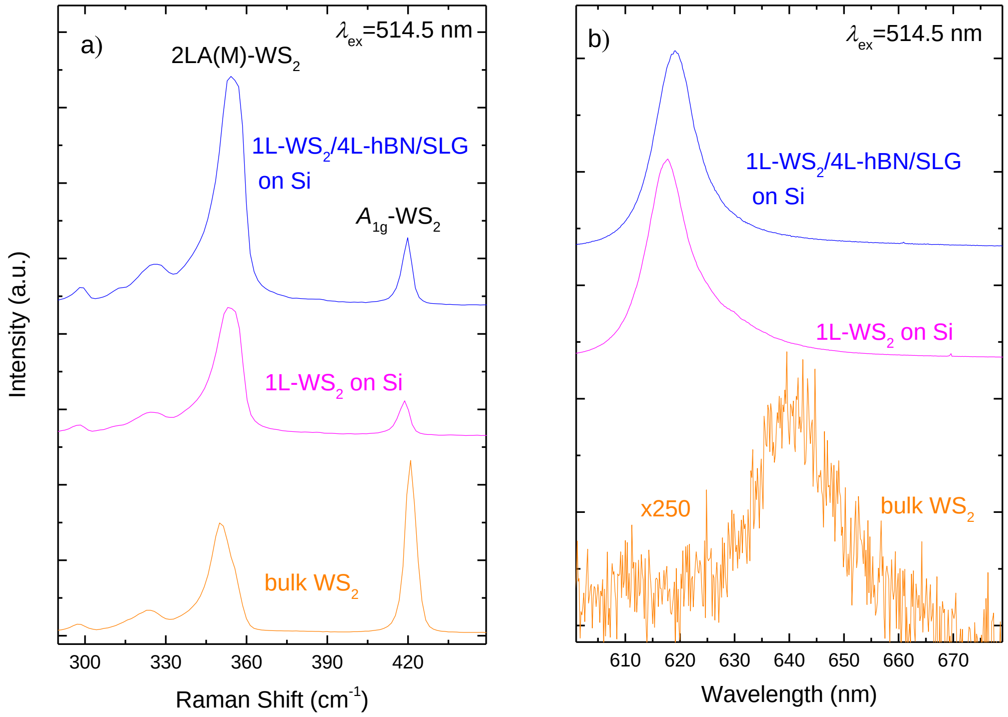

Supplementary Figure 7: Raman of hBN and SLG at different stages of WS_2 device fabrication. Comparison of (a) Raman and (b) PL spectra of bulk WS_2 (orange curve), of 1L-WS_2 on Si/SiO_2 (pink curve) and of 1L-WS_2/4L-hBN/SLG on Si/SiO_2 (blue curve).

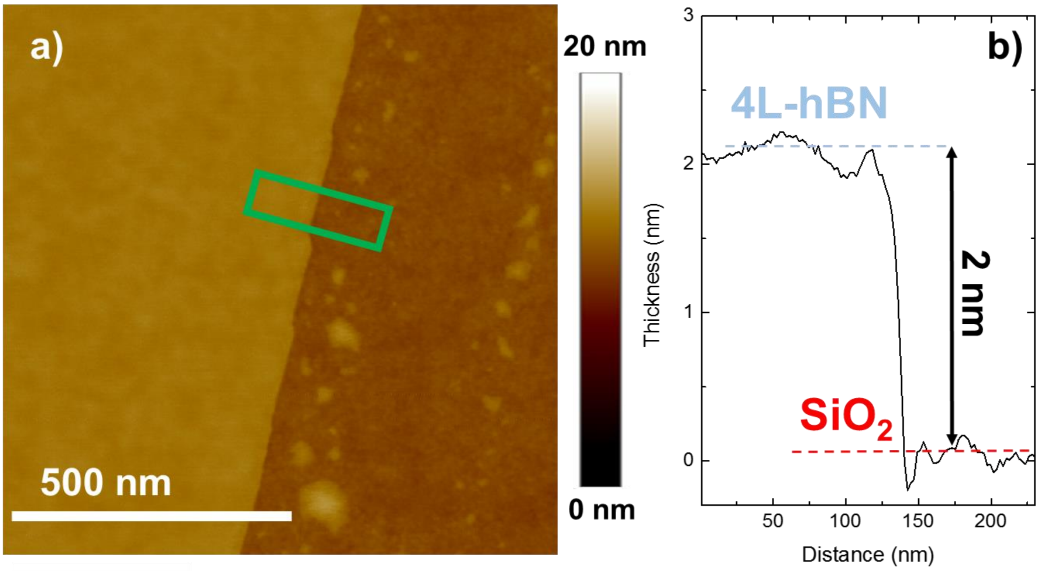

Supplementary Figure 8: Characterisation of hBN thickness. a) AFM image of hBN on SiO_2. The green rectangle shows the region where the step is measured; b) Step height ~2 nm corresponding to the area included by the green rectangle.

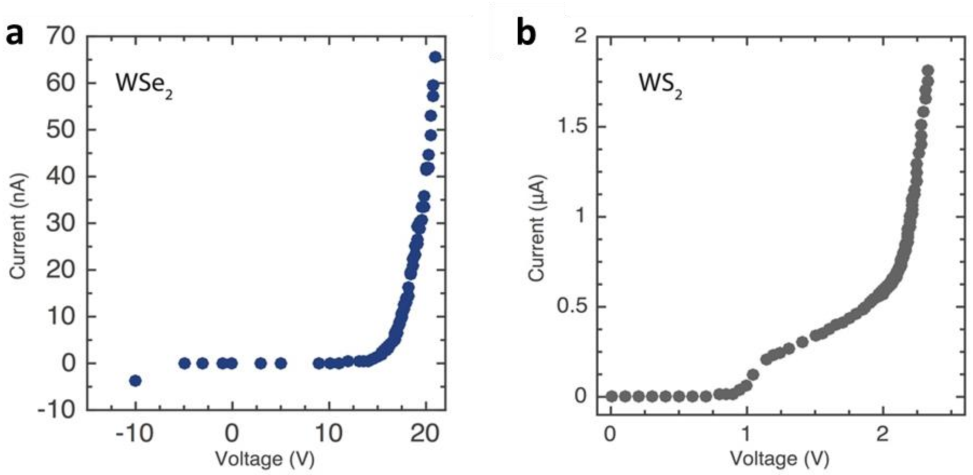

Supplementary Figure 9: Current-voltage characteristics of WSe_2 and WS_2-based QLED devices. Current vs. Voltage measurements taken at 10 K from (a) 1L-WSe_2 (a) and (b) 1L-WS_2 -based QLEDs. A negative bias applied to the SLG raises its E_F and allows electrons to tunnel into the conduction band of WSe_2, increasing the current. Similarly for the WS_2 device, by lowering the SLG E_F with a positive bias, holes can tunnel into the WS_2 valence band. The step in the I-V curve in panel b is assigned to the different current thresholds of the two 1L-WS_2 flakes present in this specific device.

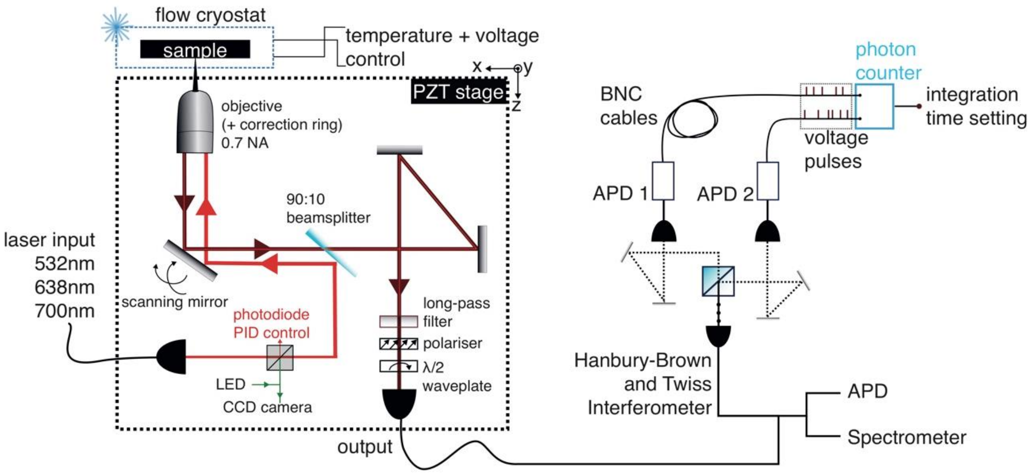

Supplementary Figure 10: Confocal microscopy setup. A home-built confocal microscope (left, enclosed by dashed lines) is used to obtain micrometre-resolved PL and EL maps. Different laser inputs are used: 638 and 700 nm for 1L and 2L-WSe_2 and 532 nm for 1L-WS_2. The charge-coupled device (CCD) camera and LED allow wide field illumination of the sample to facilitate locating the QLED on the substrate. The light output is either sent to a spectrometer or to an avalanche photodiode (APD) for PL and EL scans. For photon-correlation measurements, the output is sent to a Hanbury Brown and Twiss interferometer[30], where it is split by a 50:50 beam-splitter and two APDs. The signal from these detectors is correlated using the time-to-digital converter.

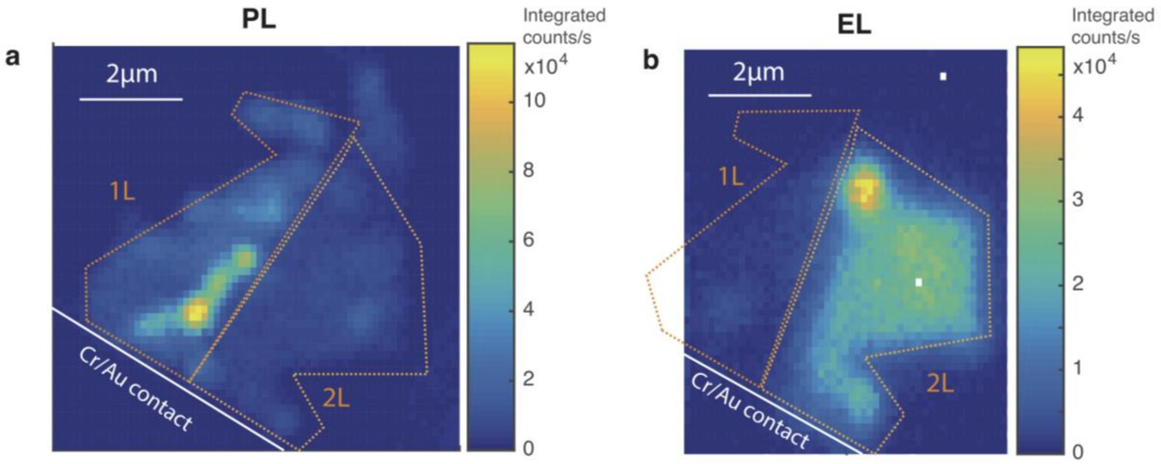

Supplementary Figure 11: Comparison of monolayer and bilayer EL emission for WSe_2-based LED. Maps of one of the WSe_2-based QLED devices taken at 10 K, showing a 1L and a 2L region which appear brighter in (a) PL and (b) EL maps respectively. One of the WSe_2-based QLED devices had an upper contact to both a monolayer and bilayer region in parallel. Interestingly, current is injected preferentially through the bilayer region, and as a result only this region lights up in EL. In contrast, the monolayer region is brighter than the bilayer in PL.

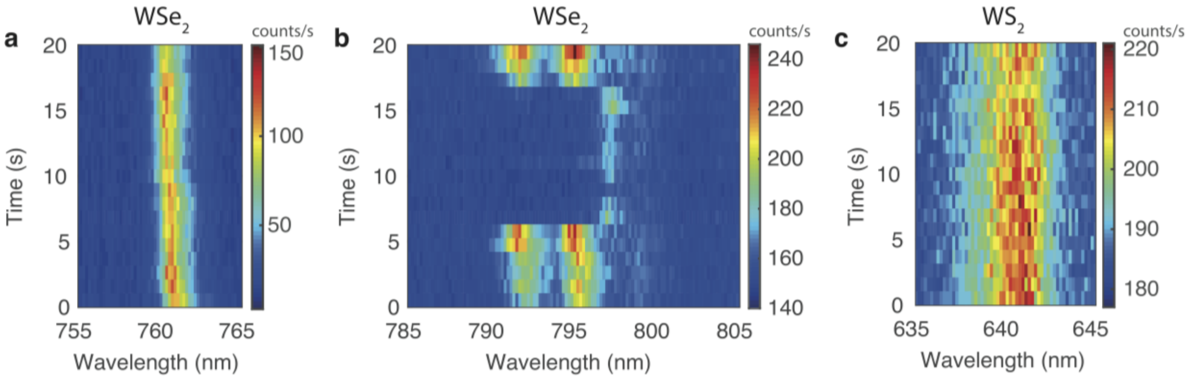

Supplementary Figure 12: Spectral wandering and blinking QLED spectra. Spectral wandering measurements of electrically-driven quantum emitters taken at 10K with a time resolution of 1s per spectra. The narrowest linewidths observed from the 1L and 2L-WSe_2-based devices are 1 nm, in contrast to those seen under PL ~0.05 nm. Measurements of the electrically driven single emitters over time show a spectral wandering ~2 nm (left panel), compared to ~0.5 – 1 nm under PL. Under EL some emitters blink on timescales of seconds, as shown in the middle panel. There appears to be no blinking at the sub-millisecond timescale. However, we observe no bunching in the photon correlation measurements, as reported previously for PL experiments on 1L and FL-WSe_2 quantum emitters[1–5]. Spectral measurements over time of the electrically driven 1L-WS_2 emitters (right panel) indicate that the spectral wandering cannot be well resolved due to a broader linewidth ~4 nm.

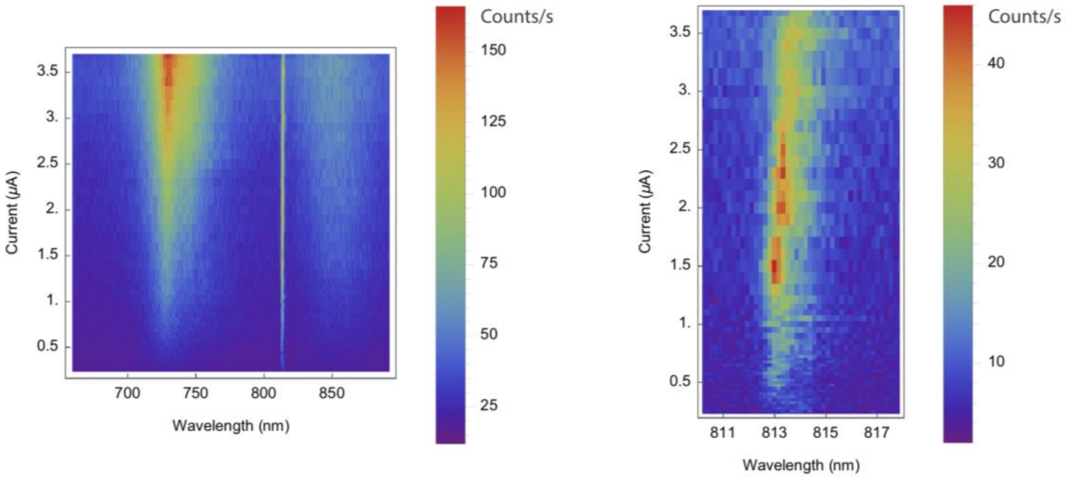

Supplementary Figure 13: WSe_2 EL spectra against injection current. (a) spectral evolution the WSe_2 unbound X^- (~730nm) and an emitter line (~814nm) against current. (b) zoom in of the emitter showing a red-shift with increasing current. This shows that it is possible to tune the emitter. However, wavelength is expected to depend on both the local carrier density and local electric field. Our present design does not allow independent control of these, since current and voltage drop across the device are linked. This makes it difficult for direct spectral tuning and to draw conclusions about the nature of the emitter. Future work will address this issue by implementing more complicated device designs that allow independent control of the parameters, with a back-gate for example.

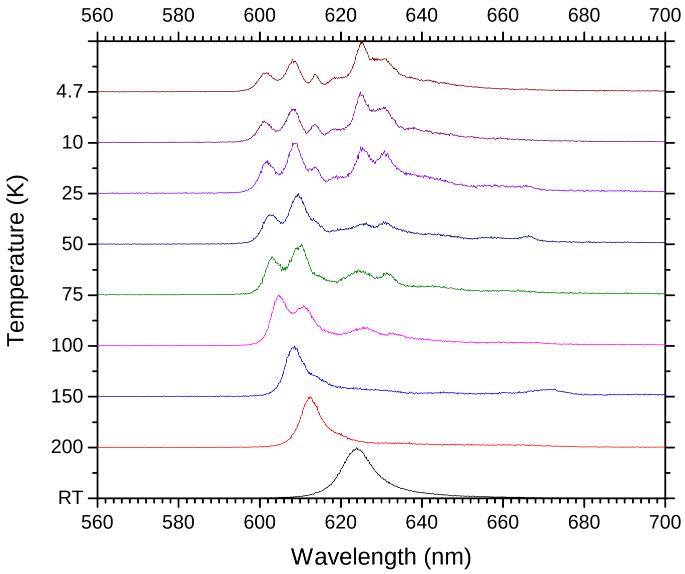

Supplementary Figure 14: WS_2 PL spectra against temperature. The temperature is the sample holder temperature. At the quoted 4.7K the sample is actually at ~10K. For the larger temperatures this discrepancy is reduced. We investigated the spectral dependence of WS_2 under PL versus temperature as shown in Figure S7._Much like in WSe_2, we see a blue-shift of unbound excitons and the appearance of a 620-640nm emission band that we attribute to localized states as the sample is cooled. This emission band coincides with the single emitter spectra shown in Fig. 3c of the main text and Fig. S8.

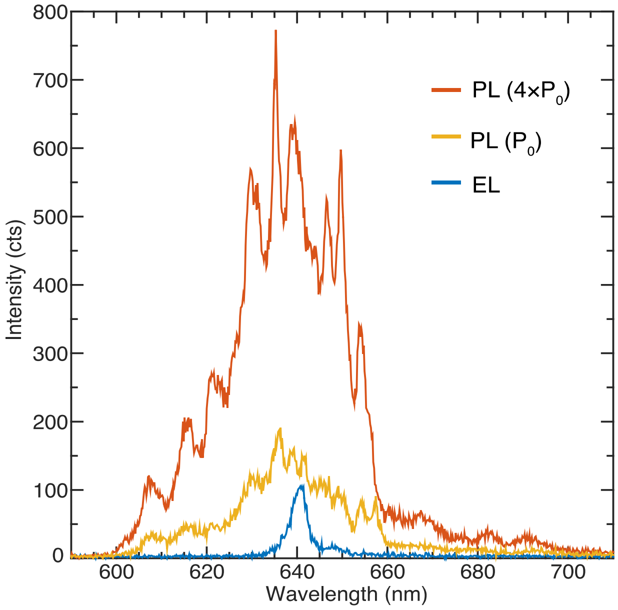

Supplementary Figure 15: Comparison of low temperature PL and EL spectra for WS_2-based QLED. EL and PL from the 1L-WS_2-based device at the location where single-photon emission is observed. P_0 is 225 nW for the PL spectra and the injected current is 5754 nA (1.985 V) for the EL spectrum. Fig. S8 compares the spectra taken in EL and PL at 10K from the 1L-WS_2-based QLED, at the site were single-photon emission is seen. The PL spectrum comprises multiple peaks, while the EL is narrow and predominantly a single peak. This may be due to generation of multiple exciton complexes as well as other donor-based delocalised emission from WS_2.

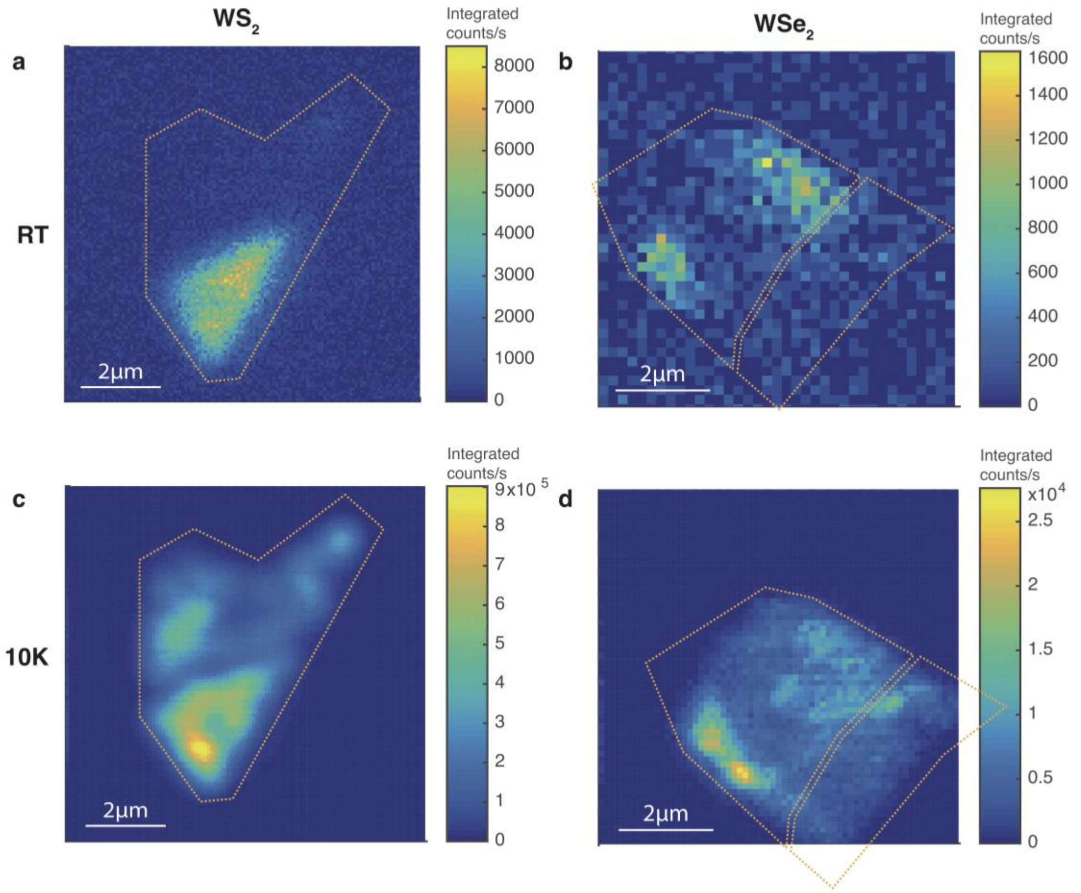

Supplementary Figure 16: Temperature-dependent EL maps. EL maps at RT and 10K of the WSe_2 and WS_2 LED devices. (a) 665 nA (1.992 V) and (c) at 665 nA (2.08 V). (b) 200 nA (-2 V) and (d) 900 nA (-3.2 V). An increase of several orders of magnitude is observed in unbound exciton EL when lowering the temperature from RT to 10 K: a 4-fold increase is measured in the 1L- and 2L-WSe_2-based LED and a ~100-fold increase in the 1L-WS_2 device. This indicates that the quantum efficiency of WS_2 is greatly enhanced in comparison to WSe_2. This could be because much of the WS_2 emission originates from the localised band, which appears at low temperatures.

Supplementary Note 1. Materials sourcing, characterization and device assembly

We measured two sets of devices. The first consists of 1L- and 2L-WSe_2 on top of hBN on top of SLG on Si/SiO_2. We use 2L-WSe_2 in addition to 1L- to compare SPE in the two cases, as discussed in the main text. The second set has the same architecture but uses 1L-WS_2 instead of WSe_2. The crystals and heterostructures are characterised at room temperature using a combination of optical contrast, AFM, Raman spectroscopy and photoluminescence.

Optical images are acquired using a Nikon Eclipse optical microscope equipped with a 100x objective (numerical aperture 0.85). If no filter is specifically mentioned, a white light is used. AFM images are acquired using a Bruker Dimension Icon microscope in PeakForce Tapping mode. Raman and PL Spectra are acquired using a Renishaw inVia micro-spectrometer (resolution pixel-to-pixel~1.2 cm^{-1}), a 100x objective (numerical aperture 0.9)

and a spot size ~1 µm. All spectra are recorded in back-scattering at 514.5 nm. The power is kept below 100 µW to prevent heating effects.

Supplementary Note 2. WSe_2/hBN/SLG heterostructures

The first set of devices, based on WSe_2, are assembled in a clean room as follows.

Highly oriented pyrolytic graphite (HOPG) sourced from NGS Naturgrafit is exfoliated by micromechanical cleavage[6,7] with adhesive tape (silicone-free, Ultron) and deposited on oxidised silicon wafers (oxide thickness 285 nm) to ensure good visibility[8]. SLG flakes are identified by optical contrast (Supplementary Fig. 1)[8]. Optical contrast is calculated as $1-I_c/I_s$, where I_c is the intensity of light reflected by the flake as measured by the CCD, and I_s is the intensity of the light reflected by the substrate. In the green channel of the CCD camera, where contrast is maximum for SLG on the specific SiO_2 thickness, the optical contrast of SLG is ~6%. SLG is used as the bottom layer in the heterostructure, in contact with the Si/SiO_2 substrate on top of which it was exfoliated.

In order to build a heterostructure with clean interfaces, it is crucial to assemble the layers as soon as possible after the flakes are exfoliated. Therefore after optical contrast analysis, further characterisation is only performed after the full heterostructure is assembled.

After the exfoliation and identification of SLG, the second step consists in fabricating FL-hBN. We start from bulk hBN single crystals grown by the temperature-gradient method under high pressure and high temperature, as discussed in the main text[9].

Before exfoliation, bulk hBN crystals are characterised by Raman spectroscopy, as shown in Supplementary Fig. 2a (orange line). The peak at ~1365.5 cm^{-1} corresponds to the E_{2g} mode of bulk hBN[10–12]. Its full width at half maximum *FWHM* is ~9.2 cm^{-1}. The *FWHM* of hBN is linked to its crystal size according to the following equation: $FWHM=1417/L_a+8.7$[10], where L_a is the hBN crystal size in Angstroms. In our case, this corresponds to an in plane average grain size of at least 200 nm[10].

FL-hBN flakes are prepared via micromechanical cleavage of the bulk hBN on oxidised Si wafers (SiO_2 285 nm thick). After exfoliation, FL-hBN are identified on the Si/SiO_2 substrate by optical contrast. Optical images are acquired using a filter at 580 nm to select the incident wavelength. In these conditions, the optical contrast of 1L-hBN on Si/SiO_2 is highest, ~2%, and it increases linearly with the number of layers. Supplementary Fig. 3a shows the optical contrast of FL-hBN exfoliated on Si/SiO_2 measured under these conditions. ~10%, corresponds to a 5L-BN.

Bulk $WSe_{2,}$ sourced from HQgraphene, is characterized prior to exfoliation by Raman spectroscopy and PL. The Raman spectrum of bulk WSe_2 is shown in Supplementary Fig. 4a (orange). The main peak at ~250 cm^{-1} is the convolution of the A_{1g} and E_{2g} modes of WSe_2 at ~247 and ~251 cm^{-1} respectively[13], and the shoulder at ~260 cm^{-1} belongs to the 2LA(M) mode[14]. The ~4 cm^{-1} distance between A_{1g} and E_{2g} and the ratio between the intensity of the E_{2g} and 2LA(M) mode, I(E_{2g}-WSe_2)/I(2LA(M) E_{2g}-WSe_2)~1.5, are consistent with the reported spectrum of bulk WSe_2[13]. PL from bulk WSe_2 crystals is shown in Supplementary Fig. 4b (orange curve). The peak at ~890 nm corresponds to the optical bandgap of bulk WSe_2[15]. Bulk WSe_2 is then exfoliated by micromechanical cleavage on oxidised silicon wafers (oxide 285 nm thick) following the same procedures as for hBN and graphite. Single-

layers are identified via optical contrast using the green channel, as for Supplementary Fig. 3b. The contrast of 1L-WSe_2 is significantly higher than both SLG and hBN, ~25%.

After having exfoliated and identified the separate crystals, the heterostructure is assembled via a dry-transfer technique[7,16]: a transparent stack comprising a glass slide, a polydimethylsiloxane (PDMS) layer (~1-mm thick) attached to the glass and polycarbonate (PC) as external film, of roughly the same size of PDMS, is mounted on a micromanipulator positioned under an optical microscope with a temperature-controlled stage. The materials forming the stack are all transparent, which allows the visualization of the sample below. The Si/SiO_2 substrate supporting the 1L-WSe_2 flake is placed on the stage and is the first to be picked up, as it will form the top layer of the final structure. After adjusting the alignment between the stack and the 1L-WSe_2 crystal, the stage is heated to ~50 °C, then the transfer stack is brought into contact with the crystal. Under these conditions, crystals can be picked up on the stack due to their higher adhesion to PC compared to SiO_2. The substrate is then changed and another Si/SiO_2 substrate with 5L-hBN is placed on the stage. The procedure is repeated: the WSe_2 on PC/PMMS/glass is aligned to the hBN crystal. Then the two crystals are brought in contact and finally 5L-hBN can be picked up to form a 1L-WSe_2/5L-hBN layer on the supporting stack.

1L-WSe_2 and 5L-hBN adhere strongly to each other. When parts of hBN stick out of the WSe_2 layer, the adhesion of 5L-hBN to PC at~50 °C is still enough to pick up the whole stack without damage. Finally, the Si/SiO_2 substrate with the selected SLG flakes is placed on the stage. The 1L-WSe_2/5L-hBN layer on the transfer stack is then aligned to the SLG flake on Si/SiO_2 and all the layers are brought in contact. The temperature is raised to ~100 °C, which ensures adhesion of the whole PC film to SiO_2. The PC can therefore be released from the PDMS/glass. Then, the sample is soaked in chloroform to dissolve the PC film, leaving the final heterostructure. This is then characterised by Raman spectroscopy on different points: on an area comprising only SLG on Si/SiO_2, on an area comprising only 5L-hBN on Si/SiO_2, on an area comprising only 1L-WSe_2 on Si/SiO_2, on an area formed only by 5L-hBN/SLG and on the full 1L-WSe_2/5L-hBN/SLG stack.

Supplementary Fig. 2 (black curve), plots the Raman spectrum of a SLG on Si/SiO_2. The G peak corresponds to the high frequency E_{2g} phonon at Γ[17]. The D peak is due to the breathing modes of six-atom rings and requires a defect for its activation[17,18]. It comes from transverse optical (TO) phonons around the Brillouin Zone (BZ) edge K[17], is active by double resonance (DR)[19] and is strongly dispersive with excitation energy due to a Kohn Anomaly (KA) at K[20]. DR can also happen as intra-valley process, i.e. connecting two points belonging to the same cone around K or K'. This gives the so-called D' peak. The 2D peak is the D peak overtone while the 2D' peak is the D' overtone. Since 2D and 2D' originate from a process where momentum conservation is satisfied by two phonons with opposite wave vectors, no defects are required for their activation, and are thus always present[21]. The 2D peak is a single Lorentzian in SLG, whereas it splits into several components as the number of layers increases, reflecting the evolution of the electronic band structure[22]. The 2D peak in Supplementary Fig. 1b is a single Lorentzian, which confirms the SLG nature of the sample. The position of the G peak, *Pos*(G), is ~1591 cm^{-1}, its full width at half maximum, *FWHM*(G), ~8.5 cm^{-1}, *Pos*(2D) ~2685 cm^{-1}, *FWHM*(2D)~28.7 cm^{-1}, the intensity ratio between 2D and G peak, *I*(2D)/*I*(G), ~1.17 and area ratio, *A*(2D)/*A*(G), ~3.9. This allows us

to estimate a doping ~0.8x10^{13} cm^{-2}, corresponding to a Fermi level ~370 meV[23]. The absence of D peak indicates negligible defect density[18,24,25].

The Raman spectrum of the 5L-hBN on SiO_2 is shown Supplementary Fig. 2a (red line). The E_{2g} peak is at ~1367.5 cm^{-1}, ~2 cm^{-1} blueshifted compared to the bulk, consistent with what expected from a thinner crystal[10,12], while *FWHM* (E_{2g}-5L-hBN) is ~10.5 cm^{-1}, 0.1 cm^{-1} higher than the error bar introduced by the resolution of the spectrometer, which corresponds to a grain size ~80 nm[10]. Raman and PL spectra of 1L-WSe_2 on Si/SiO_2 (magenta) are shown in Supplementary Fig. 4. The peak at ~250 cm^{-1} belongs to the A_1' and E' modes[13,14], which are degenerate in 1L-WSe_2[13]. I(E_{2g}-1L-WSe_2)/I(2LA(M)-1L-WSe_2) increases to ~10, consistent with a low number of layers[13]. The absence of the A^2_{1g} mode at ~310 cm^{-1} is also consistent with this being 1L-WSe_2[14], however it is not advisable to use the absence of a peak as a characterization tool, because one can never be sure why something is absent[26]. So the thickness is further confirmed by PL (Supplementary Fig. 4b, magenta), where a single peak arises at ~750 nm, blueshifted ~140 nm compared to bulk WSe_2. This is due to emission from the A exciton, corresponding to the direct transition between top conduction and bottom valence band at the K and K' points[15]. The peak of 1L-WSe_2 is ~2 orders of magnitude more intense compared to the bulk crystal. No other peaks in the 800-900 nm region are seen, which would be a signature of indirect bandgap transitions of a larger number of layers[15].

Supplementary Fig. 2 (green curve) plots the Raman spectrum of 5L-hBN on SLG. *Pos*(G) is ~1590 cm^{-1}, *FWHM*(G) ~8.2 cm^{-1}, *Pos*(2D) ~2694 cm^{-1} , *FWHM*(2D) ~24.4 cm^{-1}, *I*(2D)/*I*(G) ~1.53 and *A*(2D)/*A*(G) ~4.5. We observe a ~9 cm^{-1} upshift in *Pos*(2D) compared to the SLG on SiO_2 case, while the G peak is downshifted by ~1 cm^{-1}. From these values we derive a doping ~0.3 x $10^{13}$$cm^{-2}$, reduced compared to the case of SLG on Si/SiO_2. The reduction in doping can be explained by the 5L-hBN flake covering the SLG. 5L-hBN is not only protecting SLG from the ambient air and moisture, which contribute to p-doping, but also removes moisture or other residuals on top of SLG due to a self-cleaning process[27]. *Pos* (E_{2g}-5L-hBN) ~1367.5 cm^{-1} and *FWHM* (E_{2g}-5L-hBN) ~11 cm^{-1} show no significant changes compared to the spectrum of 5L-hBN on Si/SiO_2. The D peak is absent implying no defects are introduced in SLG after placing 5L-hBN on top.

The Raman spectrum of the 1L-WSe_2/5L-hBN/SLG heterostructure is shown in Supplementary Figs. 2 and 4 (blue curves). All peaks belonging to the separate materials can be identified in the spectrum. We find *Pos*(G) ~1590 cm^{-1}, *FWHM*(G) ~8.7 cm^{-1}, *Pos*(2D) ~2695 cm^{-1}, *FWHM*(2D) ~26.2 cm^{-1}, *I*(2D)/*I*(G) ~1.58 and *A*(2D)/*A*(G) ~4.7. These values are analogous to the case of 5L-hBN on SLG and correspond to a doping of ~0.3 x10^{12} cm^{-2}. The D peak (Supplementary Fig. 1a) is still absent, implying no defects are introduced in SLG from the stacking of the layers. *Pos*(E_{2g}-5L-hBN) ~1367.5 cm^{-1}, while *FWHM* (E_{2g}-5L-hBN) ~10.5 cm^{-1}, implying no significant change in the spectrum of 5L-hBN on SLG after adding 1L-WSe_2. From the analysis of the Raman spectrum of 1L-WSe_2 on top of the stack (Supplementary Fig. 4a), *Pos*(A_1'+*E*'-1L-WSe_2) ~250 cm^{-1}, unchanged compared to the values measured on Si/SiO_2. The B exciton of 1L-WSe_2 at ~610 nm is responsible for PL background in the ~3000 cm^{-1} region of the Raman spectrum[15].

The PL spectrum of the heterostructure is shown in Supplementary Fig. 4b. The position of the A exciton remains unchanged at ~752 nm compared to the case of 1L-WSe_2

on SiO_2. In order to confirm the thickness of the 5L-hBN layer, AFM measurements are performed once the optical characterisation is concluded. AFM measurements across the hBN edge identified by optical contrast confirm the layer to be ~5 layers thick, where the thickness is ~2.4 nm. We measure the hBN interlayer step to be ~0.38 nm, which would imply a ~6 layers. However, under ambient conditions, 2d crystals on SiO_2 have been measured to be thicker than that expected by multiplying the number of layers by the interlayer distance[28]. This discrepancy is assigned the presence of a gaseous species or water intercalating between the SiO_2 and the crystal[28]. In our case, a 5L-hBN crystal should have a thickness~2 nm according to its interlayer distance, but we assume the extra ~0.5 nm to be due to the aforementioned increase in the thickness caused by the presence of contaminations.

Supplementary Note 3. WS_2/hBN/SLG heterostructures

The second set of devices are assembled and characterised as follows.

HOPG sourced from NGS Naturgrafit is exfoliated by means of micromechanical cleavage following the same procedure described in Supplementary Note 1. SLG flakes are again identified on Si/SiO_2 by optical contrast, see Supplementary Fig. 5a.

hBN is sourced and exfoliated as described in S1.1. After exfoliation, FL-hBN flakes are identified on the Si/SiO_2 by optical contrast. Supplementary Fig. 5b shows the contrast of the flake on Si/SiO_2 chosen for this device assembly, which is ~7.4%, corresponding to a 4L.

Bulk WS_2 is characterised by Raman and PL spectroscopy. The Raman spectrum of bulk WS_2 is shown in Supplementary Fig. 6a (orange curve). The most prominent peaks at ~350 and ~420 cm^{-1} are assigned to the 2LA(M) and A_{1g} modes of WS_2[29]. At 514.5 nm, the ratio between the peaks, I(2LA(M)-WS_2)/I(A_{1g}-WS_2), is a function of the number of layers and is expected to increase with decreasing number of layers[29]. In the case of bulk WS_2 the ratio is ~0.6. The PL spectrum of bulk WS_2 is shown by the orange curve, with a peak corresponding to the optical bandgap at ~640 nm. Bulk WS_2 is exfoliated on Si/SiO_2 using the same procedure as described in Supplementary Note 1. 1L-WS_2 crystals are identified by optical contrast, as shown in Supplementary Fig. 5c, where we measure a monolayer contrast ~24%.

After having exfoliated and identified the separate crystals, the heterostructure is assembled via dry-transfer with the same procedure described in S1.1.

Once the fabrication is complete, we characterise by Raman spectroscopy first the areas with the separate crystals on Si/SiO_2, then an area with 4L-hBN/SLG and finally the full stack comprising 1L-WS_2/4L-hBN/SLG. PL is also employed to further characterise WS_2 both on SiO_2 and on the heterostructure.

Supplementary Fig. 6 (black curve), plots the Raman spectrum of SLG on Si/SiO_2. The 2D peak is a single Lorentzian, which confirms the SLG nature of the sample. *Pos*(G) ~1591 cm^{-1}, *FWHM*(G) ~11.5 cm^{-1}, *Pos*(2D) ~2687 cm^{-1}, *FWHM*(2D)~31.1 cm^{-1}, I(2D)/I(G), ~1.6 and A(2D)/A(G), ~4.4 indicate doping ~$0.5x10^{13}$. The absence of a D peak indicates negligible defects. The Raman spectrum of the 4L-hBN on Si/SiO_2 is shown in Supplementary Fig. 6a (red curve). *Pos* (E_{2g}-4L-hBN) is ~1367 cm^{-1}, ~1.5 cm^{-1} blueshifted compared to the bulk crystal and consistent with a low number of layers[12]. *FWHM* (E_{2g}-4L-

hBN)~9.3 cm^{-1} is analogous to the bulk crystal and corresponds to a grain size >200 nm. The Raman spectrum of 1L-WS_2 on Si/SiO_2 is shown in Supplementary Fig. 7a (pink curve). The 2LA(M) and A_{1g} modes are respectively at ~353 and ~419 cm^{-1}. *I*(2LA(M)-1L-WS_2)/*I*(A_{1g}-1L-WS_2) is~2.6, over 4 times higher compared to the bulk case (~0.6). This is a signature of a monolayer, because a 2L-WS_2 is expected to have *I*(2LA(M)-2L-WS_2)/*I*(A_{1g}-2L-WS_2)~1[29]. In order to further confirm the thickness of the exfoliated 1L-WS_2, its PL spectrum is acquired, Supplementary Fig. 6b (pink curve). The main feature at ~618 nm, ~20 nm blueshifted compared to the bulk case, corresponds to emission from the A exciton, corresponding to the direct optical bandgap between the top valence and the bottom conduction band of 1L-WS_2. Furthermore, the intensity is ~250 times higher compared to the bulk case, as expected[15].

Supplementary Fig. 6 (green curve) plots the Raman spectrum of 4L-hBN on SLG. *Pos*(G) ~1593 cm^{-1}, *FWHM*(G) ~14.5 cm^{-1}, *Pos*(2D) ~2699.5 cm^{-1}, *FWHM*(G)~14.5 cm^{-1}, *I*(2D)/*I*(G), ~1.91
and *A*(2D)/*A*(G) ~4.45. This indicates doping~0.4x10^{13}. No D peak is seen. *Pos* (E_{2g}-4L-hBN)~1365.5 cm^{-1}, *FWHM*(E_{2g}-4L-hBN) is ~11.8 cm^{-1}, ~2.5 cm^{-1} broader compared to the case of 4L-hBN on SLG, indicating a smaller grain size.

We then perform Raman and PL characterisation on the whole 1L-WS_2/4L-hBN/SLG heterostructure, as shown in Supplementary Figs. 6 and 7 (blue curves). *Pos*(G) ~1591.5 cm^{-1}, *FWHM*(G) ~15.3 cm^{-1}, *Pos*(2D) ~2693.5 cm^{-1}, *FWHM*(2D) ~38.5 cm^{-1}, *I*(2D)/*I*(G) ~1.9 and *A*(2D)/*A*(G) ~2.3. This indicates doping~0.3x10^{13}. *Pos* (E_{2g}-4L-hBN) ~1366.5 cm^{-1}, and *FWHM*(E_{2g}-4L-hBN) ~11 cm^{-1}. *Pos*(2LA(M)-WS_2) ~353 cm^{-1}, *Pos*(A_{1g}-WS_2) ~419 cm^{-1}, with no change compared to 1L-WSe_2 characterised on Si/SiO_2. Supplementary Fig. 7b, blue line, shows the PL spectrum of the 1L-WS_2/4L-hBN/ SLG heterostructure. The A exciton at ~619 nm is nearly unchanged compared to the PL spectrum of 1L-WS_2 on Si/SiO_2.

As a last step we perform AFM characterisation to confirm the thickness derived from optical contrast, as shown in Supplementary Fig. 8. The step between 4L-hBN and SiO_2 is ~2 nm. As discussed in Supplementary Note 1, considering an interlayer distance ~0.38 nm and an increase in thickness due to the effect of the environment ~0.5 nm, we conclude that the flake is a 4L-hBN.

Supplementary References

1. Srivastava, A. *et al.* Optically active quantum dots in monolayer WSe_2. *Nat. Nanotechnol.* **10,** 491–496 (2015).
2. He, Y.-M. *et al.* Single quantum emitters in monolayer semiconductors. *Nat. Nanotechnol.* **10,** 497–502 (2015).
3. Chakraborty, C., Kinnischtzke, L., Goodfellow, K. M., Beams, R. & Vamivakas, A. N. Voltage-controlled quantum light from an atomically thin semiconductor. *Nat. Nanotechnol.* **10,** 507–511 (2015).
4. Koperski, M. *et al.* Single photon emitters in exfoliated WSe_2 structures. *Nat. Nanotechnol.* **10,** 503–506 (2015).
5. Tonndorf, P. *et al.* Single-photon emission from localized excitons in an atomically thin semiconductor. *Optica* **2,** 347 (2015).
6. Novoselov, K. S. *et al.* Two-dimensional atomic crystals. *Proc. Natl. Acad. Sci. U. S. A.* **102,** 10451–3 (2005).

7. Bonaccorso, F. *et al.* Production and processing of graphene and 2d crystals. *Mater. Today* **15,** 564–589 (2012).
8. Casiraghi, C. *et al.* Rayleigh imaging of graphene and graphene layers. *Nano Lett.* **7,** 2711–2717 (2007).
9. Watanabe, K., Taniguchi, T. & Kanda, H. Direct-bandgap properties and evidence for ultraviolet lasing of hexagonal boron nitride single crystal. *Nat. Mater.* **3,** 404–409 (2004).
10. Nemanich, R. J., Solin, S. A. & Martin, R. M. Light scattering study of boron nitride microcrystals. *Phys. Rev. B* **23,** 6348–6356 (1981).
11. Reich, S. *et al.* Resonant Raman scattering in cubic and hexagonal boron nitride. *Phys. Rev. B* **71,** 205201 (2005).
12. Arenal, R. *et al.* Raman spectroscopy of single-wall boron nitride nanotubes. *Nano Lett.* **6,** 1812–6 (2006).
13. Terrones, H. *et al.* New first order Raman-active modes in few layered transition metal dichalcogenides. *Sci. Rep.* **4,** 4215 (2014).
14. Zhao, W. *et al.* Lattice dynamics in mono- and few-layer sheets of WS_2 and WSe_2. *Nanoscale* **5,** 9677–83 (2013).
15. Zhou, B. *et al.* Evolution of electronic structure in Atomically Thin Sheets of WS_2 and WSe_2. *ACS Nano* **7,** 791–797 (2013).
16. Zomer, P. J., Guimarães, M. H. D., Brant, J. C., Tombros, N. & van Wees, B. J. Fast pick up technique for high quality heterostructures of bilayer graphene and hexagonal boron nitride. *Appl. Phys. Lett.* **105,** 013101 (2014).
17. Tuinstra, F. Raman Spectrum of Graphite. *J. Chem. Phys.* **53,** 1126 (1970).
18. Ferrari, A. C. & Robertson, J. Interpretation of Raman spectra of disordered and amorphous carbon. *Phys. Rev. B* **61,** 14095–14107 (2000).
19. Thomsen, C. & Reich, S. Double resonant raman scattering in graphite. *Phys. Rev. Lett.* **85,** 5214–7 (2000).
20. Piscanec, S., Lazzeri, M., Mauri, F., Ferrari, A. C. & Robertson, J. Kohn anomalies and electron-phonon interactions in graphite. *Phys. Rev. Lett.* **93,** 185503 (2004).
21. Basko, D. M., Piscanec, S. & Ferrari, A. C. Electron-electron interactions and doping dependence of the two-phonon Raman intensity in graphene. *Phys. Rev. B* **80,** 165413 (2009).
22. Ferrari, A. C. *et al.* Raman Spectrum of Graphene and Graphene Layers. *Phys. Rev. Lett.* **97,** 187401 (2006).
23. Das, A. *et al.* Monitoring dopants by Raman scattering in an electrochemically top-gated graphene transistor. *Nat. Nanotechnol.* **3,** 210–215 (2008).
24. Bruna, M. *et al.* Doping dependence of the Raman spectrum of defected graphene. *ACS Nano* **8,** 7432–41 (2014).
25. Cançado, L. G. *et al.* Quantifying defects in graphene via Raman spectroscopy at different excitation energies. *Nano Lett.* **11,** 3190–3196 (2011).
26. Ferrari, A. C. & Basko, D. M. Raman spectroscopy as a versatile tool for studying the properties of graphene. *Nat. Nanotechnol.* **8,** 235–246 (2013).
27. Haigh, S. J. *et al.* Cross-sectional imaging of individual layers and buried interfaces of graphene-based heterostructures and superlattices. *Nat. Mater.* **11,** 764–7 (2012).
28. Ishigami, M., Chen, J. H., Cullen, W. G., Fuhrer, M. S. & Williams, E. D. Atomic structure of graphene on SiO_2. *Nano Lett.* **7,** 1643–8 (2007).
29. Berkdemir, A. *et al.* Identification of individual and few layers of WS_2 using Raman Spectroscopy. *Sci. Rep.* **3,** 1755 (2013).
30. Hanbury Brown, R. & Twiss, R. Q. A Test of a New Type of Stellar Interferometer on Sirius. *Nature* **178,** 1046–1048 (1956).
